# Dance and Music in Children’s Literature: A Qualitative Study of Intergenerational Solidarity Themes

**DOI:** 10.1093/geroni/igaa057.022

**Published:** 2020-12-16

**Authors:** Stephen Fogle

**Affiliations:** University of Nebraska Omaha, Omaha, Nebraska, United States

## Abstract

Intergenerational Solidarity is consistently recognized by the United Nations as a primary focus for work being undertaken to build a society for all ages. This study utilized a qualitative methodology to examine themes of intergenerational solidarity contained within children’s literature. Specifically, this study explored intergenerational examples of dance and music shared by older adults and children. McGuire’s (2016) Growing Up and Growing Older annotated bibliography for preschool-to-third grade children, which contains over seventy pages of non-ageist children’s literature references (N= 411), served as the sample frame for this study. A sample of six story and picture books was selected after inclusion criteria and availability from two public children’s libraries considerations were met. Inclusiveness of the present sample is manifested through geographic origin of dance and music traditions as well as the age range, gender, primary spoken language, and kin relationships of the older adult and children characters. Results revealed three intergenerational solidarity themes: 1) a humanizing portrayal of an older adult, 2) common cause, 3) continuity of tradition. This study demonstrates the efficacy of the arts, specifically dance and music, for facilitating intergenerational solidarity. This study identifies three themes that primary school teachers and children's librarians can utilize when selecting reading material about intergenerational solidarity. Finally, this study contributes to decades of pioneering educational gerontology literature focused on combating ageism through development of curricula that stimulate discovery of the elder within.

